# Johannesburg Hospital Board's Experiment

**Published:** 1920-09-18

**Authors:** 


### Johannesburg Hospital Board's Experiment.

In South Africa the problem of poverty is complicated by the natives. Domestic servants as a rule are young male natives, and one of their undesirable traits is that of being "death on" crockery. Crockery breakages form a considerable item of wear-and-tear expenditure of private houses, hotels, and, last but not least, hospitals. In this connection it is illuminating to learn from Johannesburg that so costly had been the crockery breakages at the General Hospital there that the Hospital Board has, on the advice of the medical superintendent, decided to offer to each of the natives engaged at the hospital in washing dishes a premium of 5s. on his monthly wages, and, if anything is broken, the cost is to be deducted from his pay.

The hope is that the premium will encourage "boys" —as natives are called in South Africa—to be less careless. It is believed that the £6 10s. which the scheme will cost the hospital per month will be more than saved by the premium.

A MOVEMENT is on foot to provide hospital accommodation for Aberdeen Cape Province.

